# 4-Amino-*N*-(4,6-dimethyl­pyrimidin-2-yl)benzene­sulfonamide–benzoic acid (1/1)

**DOI:** 10.1107/S1600536810034094

**Published:** 2010-08-28

**Authors:** Hadi D. Arman, Trupta Kaulgud, Edward R. T. Tiekink

**Affiliations:** a Department of Chemistry, The University of Texas at San Antonio, One UTSA Circle, San Antonio, Texas 78249-0698, USA; b Department of Chemistry, University of Malaya, 50603 Kuala Lumpur, Malaysia

## Abstract

The constituents of the title co-crystal, C_12_H_14_N_4_O_2_S·C_7_H_6_O_2_, are connected by an eight-membered hetero-synthon {⋯NCNH⋯OCOH}, whereby the carb­oxy­lic acid forms donor and acceptor hydrogen bonds with a pyrimidine N atom and the adjacent amine, respectively. The dimeric aggregates thus formed are arranged in rows with their terminal NH_2_ groups forming N—H⋯O hydrogen bonds with neighbouring aggregates to form a two-dimensional array in the *ac* plane with an overall T-shaped topology. Layers inter­digitate along the *b* axis being connected by C—H⋯O, C—H⋯π and π–π [centroid–centroid distance = 3.6316 (19) Å] inter­actions.

## Related literature

For related studies on co-crystal formation, see: Broker & Tiekink (2007 ▶); Ellis *et al.* (2009 ▶); Arman *et al.* (2010 ▶). For related structures of carb­oxy­lic acids with 4-amino-*N*-(4,6-dimethyl­pyrimidin-2-yl)benzene-1-sulfonamide, see: Caira (1991 ▶, 1992 ▶).
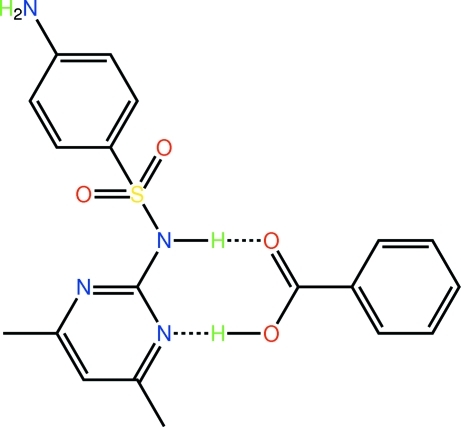


## Experimental

### 

#### Crystal data

C_12_H_14_N_4_O_2_S·C_7_H_6_O_2_
*M* *_r_* = 400.45
Orthorhombic, 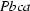

*a* = 15.203 (6) Å
*b* = 14.006 (5) Å
*c* = 18.015 (7) Å
*V* = 3836 (2) Å^3^
*Z* = 8
Mo *K*α radiation
μ = 0.20 mm^−1^
*T* = 98 K
0.35 × 0.23 × 0.10 mm

#### Data collection

Rigaku AFC12/SATURN724 diffractometer
Absorption correction: multi-scan (*ABSCOR*; Higashi, 1995 ▶) *T*_min_ = 0.828, *T*_max_ = 1
30274 measured reflections
4404 independent reflections
4137 reflections with *I* > 2σ(*I*)
*R*_int_ = 0.073

#### Refinement

*R*[*F* ^2^ > 2σ(*F* ^2^)] = 0.065
*wR*(*F* ^2^) = 0.159
*S* = 1.17
4404 reflections
267 parameters
5 restraints
H atoms treated by a mixture of independent and constrained refinement
Δρ_max_ = 0.41 e Å^−3^
Δρ_min_ = −0.55 e Å^−3^

### 

Data collection: *CrystalClear* (Molecular Structure Corporation & Rigaku, 2005 ▶); cell refinement: *CrystalClear*; data reduction: *CrystalClear*; program(s) used to solve structure: *SHELXS97* (Sheldrick, 2008 ▶); program(s) used to refine structure: *SHELXL97* (Sheldrick, 2008 ▶); molecular graphics: *ORTEP-3* (Farrugia, 1997 ▶) and *DIAMOND* (Brandenburg, 2006 ▶); software used to prepare material for publication: *publCIF* (Westrip, 2010 ▶).

## Supplementary Material

Crystal structure: contains datablocks global, I. DOI: 10.1107/S1600536810034094/hg2707sup1.cif

Structure factors: contains datablocks I. DOI: 10.1107/S1600536810034094/hg2707Isup2.hkl

Additional supplementary materials:  crystallographic information; 3D view; checkCIF report

## Figures and Tables

**Table 1:** Hydrogen-bond geometry (Å, °) *Cg*1 is the centroid of the C13–C18 ring.

*D*—H⋯*A*	*D*—H	H⋯*A*	*D*⋯*A*	*D*—H⋯*A*
O4—H4o⋯N4	0.85 (2)	1.79 (2)	2.639 (3)	177 (3)
N2—H3n⋯O3	0.89 (2)	1.90 (2)	2.787 (3)	176 (3)
N1—H1n⋯O1^i^	0.89 (2)	2.07 (2)	2.952 (3)	173 (3)
N1—H2n⋯O3^ii^	0.88 (2)	2.31 (3)	3.073 (3)	144 (2)
C12—H12c⋯O1^iii^	0.98	2.58	3.455 (3)	149
C11—H11c⋯*Cg*1^iv^	0.98	2.76	3.672 (3)	155

Symmetry codes: (i) 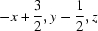; (ii) 
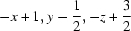
; (iii) 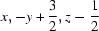; (iv) 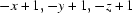.

**Fig. 1.**
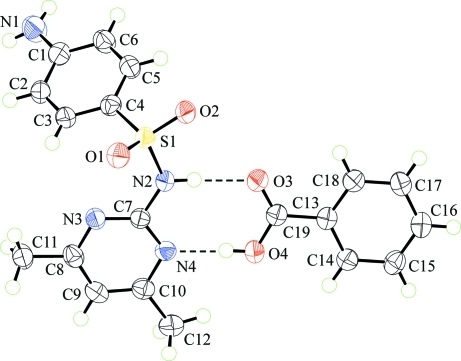
Molecular structure of the constituents of co-crystal (I) showing atom-labelling scheme and displacement ellipsoids at the 70% probability level. The O—H···N and N—H···O hydrogen bonds are shown as dashed lines.

**Fig. 2.**
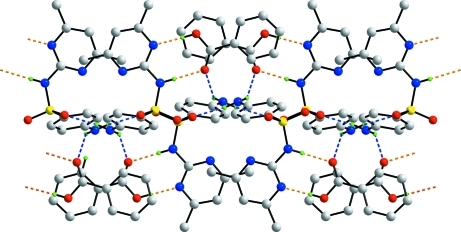
View of the supramolecular layer in projection down the b axis highlighting the rows of benzoic acid and pyrimidine residues connected via {···NCNH···OCOH} synthons (orange dashed lines). The amino-H···O hydrogen bonds are shown as blue dashed lines. Hydrogen atoms not involved in hydrogen bonding have been omitted for reasons of clarity.

**Fig. 3.**
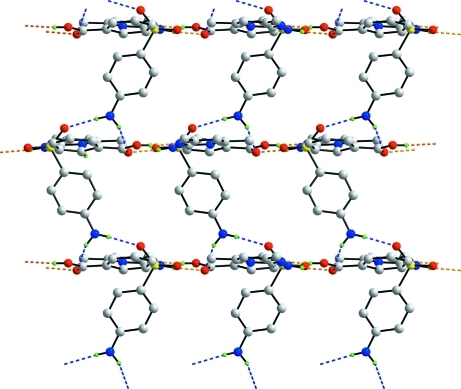
Side-on view of the projection shown in Fig. 2 highlighting the two-dimensional array. Colour code for hydrogen bonds and atom omissions as for Fig. 2.

**Fig. 4.**
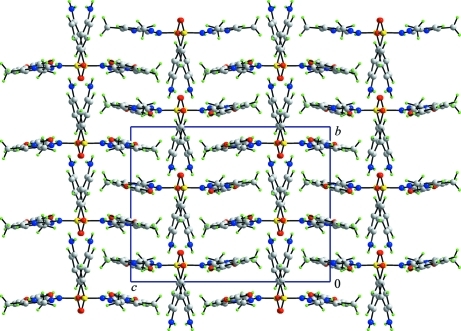
Unit-cell contents of (I) shown in projection down the a axis, highlighting the inter-digitation of rows of T-shaped and inverted T-shaped molecules.

## supplementary crystallographic information

### Comment

In continuation of co-crystallization experiments of molecules related to pharmaceuticals (Broker & Tiekink, 2007; Ellis *et al.*, 2009; Arman *et al.*, 2010), the title co-crystal containing a 1:1 ratio of 4-amino-*N*-(4,6-dimethylpyrimidin-2-yl)benzene-1-sulfonamide and benzoic acid was isolated, (I). Co-crystals of the sulfonamide with various substituted benzoic acid derivatives have been investigated previously (Caira, 1991; Caira, 1992).

A single molecule of each component comprises the asymmetric unit of (I), Fig. 1. These are connected into dimeric aggregates by an eight membered hetero-synthon {···NCNH···OCOH} involving the O3-carboxylic acid-H donating to the pyrimidine-N4 and the carbonyl-O4 accepting a hydrogen bond from the adjacent N2-amine-H. Such synthons are common to related co-crystals (Caira, 1991; Caira, 1992).

In the crystal packing, the benzoic acid and pyrimidine residues lie parallel to the *ac* plane and are arranged in a row along the *a* axis as highlighted in Fig. 2. The sulfonamide-N1-amine-H atoms bridge successive dimeric aggregates of an adjacent row. This occurs by the formation of hydrogen bonds to the carbonyl-O3 of one dimeric aggregate and a second N–H···O interaction involving the sulfonamide-O1 atom of another. This establishes a two-dimensional array, Fig. 3, that has an overall T-shaped topology. As shown in Fig. 4, the global crystal packing comprises the inter-digitation of successive rows of T-shaped and inverted T-shaped molecules. The interactions between the inter-digitated residues are of the type C—H···O and C—H···π, Table 1, and π–π [*Cg*(N3,N4,C7—C10)···*Cg*(C13—C18) = 3.6316 (19) Å for *i*: 1/2 + *x*, 11/2 - *y*, 1 - *z*].

### Experimental

Colourless crystals of (I) were isolated from the 1/1 co-crystallization of 4-amino-*N*-(4,6-dimethylpyrimidin-2-yl)benzene-1- sulfonamide (ACROS, 0.11 mmol) and benzoic acid (ACROS, 0.11 mmol) in acetone; m. pt. 481–493 K.

### Refinement

C-bound H-atoms were placed in calculated positions (C–H 0.95–0.98 Å) and were included in the refinement in the riding model approximation with *U*_iso_(H) set to 1.2–1.5*U*_eq_(C). The N– and O-bound H-atoms were located in a difference Fourier map and were refined with distance restraints of O–H = 0.84±0.01 Å and N—H = 0.88±0.01 Å, and with *U*_iso_(H) = *xU*_eq_(carrier atom), where *x* = 1.5 for O and *x* = 1.2 for N.

### Crystal data

**Table:**

C_12_H_14_N_4_O_2_S·C_7_H_6_O_2_	*F*(000) = 1680
*M**_r_* = 400.45	*D*_x_ = 1.387 Mg m^−^^3^
Orthorhombic, *P**b**c**a*	Mo *K*α radiation, λ = 0.71069 Å
Hall symbol: -P 2ac 2ab	Cell parameters from 16577 reflections
*a* = 15.203 (6) Å	θ = 2.3–40.5°
*b* = 14.006 (5) Å	µ = 0.20 mm^−^^1^
*c* = 18.015 (7) Å	*T* = 98 K
*V* = 3836 (2) Å^3^	Block, colourless
*Z* = 8	0.35 × 0.23 × 0.10 mm

### Data collection

**Table:**

Rigaku AFC12K/SATURN724 diffractometer	4404 independent reflections
Radiation source: fine-focus sealed tube	4137 reflections with *I* > 2σ(*I*)
graphite	*R*_int_ = 0.073
ω scans	θ_max_ = 27.5°, θ_min_ = 2.3°
Absorption correction: multi-scan (*ABSCOR*; Higashi, 1995)	*h* = −19→19
*T*_min_ = 0.828, *T*_max_ = 1	*k* = −16→18
30274 measured reflections	*l* = −23→23

### Refinement

**Table:**

Refinement on *F*^2^	Primary atom site location: structure-invariant direct methods
Least-squares matrix: full	Secondary atom site location: difference Fourier map
*R*[*F*^2^ > 2σ(*F*^2^)] = 0.065	Hydrogen site location: inferred from neighbouring sites
*wR*(*F*^2^) = 0.159	H atoms treated by a mixture of independent and constrained refinement
*S* = 1.17	*w* = 1/[σ^2^(*F*_o_^2^) + (0.0647*P*)^2^ + 3.1388*P*] where *P* = (*F*_o_^2^ + 2*F*_c_^2^)/3
4404 reflections	(Δ/σ)_max_ = 0.001
267 parameters	Δρ_max_ = 0.41 e Å^−^^3^
5 restraints	Δρ_min_ = −0.55 e Å^−^^3^

### Special details

**Table:**

Geometry. All s.u.'s (except the s.u. in the dihedral angle between two l.s. planes) are estimated using the full covariance matrix. The cell s.u.'s are taken into account individually in the estimation of s.u.'s in distances, angles and torsion angles; correlations between s.u.'s in cell parameters are only used when they are defined by crystal symmetry. An approximate (isotropic) treatment of cell s.u.'s is used for estimating s.u.'s involving l.s. planes.
Refinement. Refinement of *F*^2^ against ALL reflections. The weighted *R*-factor *wR* and goodness of fit *S* are based on *F*^2^, conventional *R*-factors *R* are based on *F*, with *F* set to zero for negative *F*^2^. The threshold expression of *F*^2^ > σ(*F*^2^) is used only for calculating *R*-factors(gt) *etc*. and is not relevant to the choice of reflections for refinement. *R*-factors based on *F*^2^ are statistically about twice as large as those based on *F*, and *R*- factors based on ALL data will be even larger.

### Fractional atomic coordinates and isotropic or equivalent isotropic displacement parameters (Å^2^)

**Table:**

	*x*	*y*	*z*	*U*_iso_*/*U*_eq_	
S1	0.54508 (4)	0.60684 (4)	0.73129 (3)	0.02568 (16)	
O1	0.60107 (11)	0.68686 (12)	0.74705 (9)	0.0323 (4)	
O2	0.45840 (11)	0.60392 (12)	0.76309 (9)	0.0310 (4)	
O3	0.35026 (10)	0.58510 (12)	0.59738 (9)	0.0304 (4)	
O4	0.38399 (11)	0.61521 (13)	0.47864 (9)	0.0334 (4)	
H4O	0.4377 (9)	0.615 (2)	0.4912 (18)	0.050*	
N1	0.72042 (15)	0.23929 (16)	0.79698 (14)	0.0402 (5)	
H1N	0.7731 (10)	0.2262 (19)	0.7781 (16)	0.048*	
H2N	0.6859 (14)	0.1909 (14)	0.8089 (16)	0.048*	
N2	0.52506 (13)	0.60582 (14)	0.64115 (10)	0.0277 (4)	
H3N	0.4698 (9)	0.5961 (19)	0.6277 (15)	0.033*	
N3	0.67129 (12)	0.61923 (13)	0.60236 (10)	0.0258 (4)	
N4	0.55167 (12)	0.62139 (13)	0.51621 (10)	0.0249 (4)	
C1	0.68116 (15)	0.32487 (17)	0.78271 (12)	0.0296 (5)	
C2	0.72999 (15)	0.40339 (17)	0.75643 (12)	0.0289 (5)	
H2	0.7915	0.3965	0.7487	0.035*	
C3	0.69052 (15)	0.49023 (17)	0.74168 (12)	0.0279 (5)	
H3	0.7246	0.5424	0.7241	0.033*	
C4	0.59970 (14)	0.50071 (16)	0.75289 (12)	0.0254 (4)	
C5	0.55028 (15)	0.42415 (17)	0.78067 (12)	0.0292 (5)	
H5	0.4889	0.4315	0.7889	0.035*	
C6	0.59063 (16)	0.33828 (17)	0.79608 (13)	0.0317 (5)	
H6	0.5569	0.2873	0.8160	0.038*	
C7	0.58666 (14)	0.61564 (15)	0.58457 (12)	0.0241 (4)	
C8	0.72783 (15)	0.63113 (16)	0.54554 (13)	0.0277 (5)	
C9	0.69815 (16)	0.63989 (17)	0.47292 (13)	0.0307 (5)	
H9	0.7385	0.6494	0.4333	0.037*	
C10	0.60877 (15)	0.63456 (16)	0.45948 (12)	0.0276 (5)	
C11	0.82379 (15)	0.63460 (18)	0.56559 (14)	0.0342 (5)	
H11A	0.8301	0.6532	0.6178	0.051*	
H11B	0.8538	0.6814	0.5340	0.051*	
H11C	0.8501	0.5715	0.5581	0.051*	
C12	0.57027 (17)	0.64250 (19)	0.38306 (13)	0.0358 (5)	
H12A	0.5063	0.6351	0.3858	0.054*	
H12B	0.5949	0.5924	0.3513	0.054*	
H12C	0.5845	0.7052	0.3621	0.054*	
C13	0.23390 (14)	0.60650 (15)	0.51020 (12)	0.0253 (4)	
C14	0.21086 (15)	0.63324 (16)	0.43783 (12)	0.0268 (4)	
H14	0.2553	0.6452	0.4019	0.032*	
C15	0.12273 (15)	0.64217 (17)	0.41883 (13)	0.0300 (5)	
H15	0.1069	0.6609	0.3700	0.036*	
C16	0.05768 (16)	0.62377 (17)	0.47104 (14)	0.0320 (5)	
H16	−0.0025	0.6294	0.4576	0.038*	
C17	0.08033 (16)	0.59699 (18)	0.54334 (14)	0.0329 (5)	
H17	0.0358	0.5848	0.5791	0.039*	
C18	0.16840 (16)	0.58838 (17)	0.56251 (13)	0.0301 (5)	
H18	0.1841	0.5700	0.6115	0.036*	
C19	0.32779 (15)	0.60083 (15)	0.53280 (12)	0.0262 (4)	

### Atomic displacement parameters (Å^2^)

**Table:**

	*U*^11^	*U*^22^	*U*^33^	*U*^12^	*U*^13^	*U*^23^
S1	0.0240 (3)	0.0291 (3)	0.0240 (3)	0.0018 (2)	−0.00015 (18)	−0.00016 (19)
O1	0.0319 (9)	0.0304 (8)	0.0346 (8)	−0.0011 (7)	−0.0027 (7)	−0.0038 (7)
O2	0.0234 (8)	0.0397 (10)	0.0299 (8)	0.0061 (7)	0.0035 (6)	0.0030 (7)
O3	0.0270 (8)	0.0375 (9)	0.0268 (8)	−0.0004 (7)	−0.0025 (6)	0.0013 (7)
O4	0.0222 (8)	0.0476 (10)	0.0303 (8)	0.0011 (7)	−0.0003 (6)	0.0051 (7)
N1	0.0340 (11)	0.0322 (11)	0.0544 (13)	0.0047 (9)	0.0073 (10)	0.0061 (10)
N2	0.0212 (9)	0.0359 (10)	0.0259 (9)	−0.0016 (8)	−0.0031 (7)	0.0042 (7)
N3	0.0224 (9)	0.0256 (9)	0.0294 (9)	−0.0004 (7)	0.0004 (7)	0.0013 (7)
N4	0.0263 (9)	0.0235 (9)	0.0249 (9)	−0.0001 (7)	0.0007 (7)	0.0027 (7)
C1	0.0294 (11)	0.0307 (11)	0.0288 (10)	0.0018 (9)	−0.0012 (9)	−0.0004 (9)
C2	0.0226 (10)	0.0366 (12)	0.0275 (10)	0.0012 (9)	−0.0010 (8)	0.0002 (9)
C3	0.0240 (10)	0.0329 (11)	0.0266 (10)	−0.0035 (9)	−0.0011 (8)	0.0022 (9)
C4	0.0263 (11)	0.0276 (10)	0.0225 (9)	0.0005 (9)	−0.0015 (8)	−0.0013 (8)
C5	0.0245 (11)	0.0336 (12)	0.0295 (10)	−0.0020 (9)	0.0017 (8)	−0.0014 (9)
C6	0.0284 (11)	0.0303 (12)	0.0363 (12)	−0.0040 (9)	0.0020 (9)	0.0014 (9)
C7	0.0232 (10)	0.0223 (10)	0.0269 (10)	−0.0009 (8)	−0.0009 (8)	0.0016 (8)
C8	0.0244 (10)	0.0233 (10)	0.0354 (11)	−0.0019 (9)	0.0027 (9)	−0.0003 (9)
C9	0.0298 (11)	0.0310 (11)	0.0311 (11)	−0.0032 (9)	0.0070 (9)	0.0021 (9)
C10	0.0302 (11)	0.0241 (10)	0.0285 (10)	0.0000 (9)	0.0015 (8)	0.0006 (8)
C11	0.0239 (11)	0.0374 (13)	0.0413 (13)	−0.0013 (10)	0.0009 (9)	0.0018 (10)
C12	0.0367 (12)	0.0425 (14)	0.0280 (11)	−0.0014 (11)	0.0012 (10)	0.0046 (10)
C13	0.0251 (10)	0.0227 (10)	0.0281 (10)	0.0014 (8)	−0.0019 (8)	−0.0029 (8)
C14	0.0273 (11)	0.0262 (10)	0.0270 (10)	−0.0008 (9)	0.0000 (8)	−0.0008 (8)
C15	0.0298 (11)	0.0294 (11)	0.0308 (11)	−0.0012 (9)	−0.0075 (9)	−0.0019 (9)
C16	0.0260 (11)	0.0348 (12)	0.0353 (12)	0.0010 (9)	−0.0030 (9)	−0.0028 (10)
C17	0.0253 (11)	0.0388 (13)	0.0346 (12)	−0.0002 (10)	0.0016 (9)	0.0009 (10)
C18	0.0291 (11)	0.0331 (12)	0.0281 (11)	−0.0007 (9)	−0.0012 (9)	0.0008 (9)
C19	0.0264 (11)	0.0242 (10)	0.0280 (10)	0.0002 (8)	−0.0015 (8)	−0.0006 (8)

### Geometric parameters (Å, °)

**Table:**

S1—O1	1.4358 (18)	C6—H6	0.9500
S1—O2	1.4375 (17)	C8—C9	1.389 (3)
S1—N2	1.652 (2)	C8—C11	1.504 (3)
S1—C4	1.747 (2)	C9—C10	1.382 (3)
O3—C19	1.232 (3)	C9—H9	0.9500
O4—C19	1.313 (3)	C10—C12	1.500 (3)
O4—H4O	0.848 (10)	C11—H11A	0.9800
N1—C1	1.364 (3)	C11—H11B	0.9800
N1—H1N	0.889 (9)	C11—H11C	0.9800
N1—H2N	0.88 (2)	C12—H12A	0.9800
N2—C7	1.391 (3)	C12—H12B	0.9800
N2—H3N	0.886 (10)	C12—H12C	0.9800
N3—C7	1.327 (3)	C13—C18	1.394 (3)
N3—C8	1.347 (3)	C13—C14	1.401 (3)
N4—C7	1.344 (3)	C13—C19	1.486 (3)
N4—C10	1.353 (3)	C14—C15	1.388 (3)
C1—C2	1.409 (3)	C14—H14	0.9500
C1—C6	1.410 (3)	C15—C16	1.389 (3)
C2—C3	1.382 (3)	C15—H15	0.9500
C2—H2	0.9500	C16—C17	1.398 (3)
C3—C4	1.403 (3)	C16—H16	0.9500
C3—H3	0.9500	C17—C18	1.388 (3)
C4—C5	1.402 (3)	C17—H17	0.9500
C5—C6	1.378 (3)	C18—H18	0.9500
C5—H5	0.9500		
			
O1—S1—O2	119.14 (10)	C10—C9—C8	118.6 (2)
O1—S1—N2	108.07 (10)	C10—C9—H9	120.7
O2—S1—N2	102.86 (10)	C8—C9—H9	120.7
O1—S1—C4	109.78 (11)	N4—C10—C9	120.4 (2)
O2—S1—C4	108.84 (10)	N4—C10—C12	116.9 (2)
N2—S1—C4	107.40 (10)	C9—C10—C12	122.7 (2)
C19—O4—H4O	115 (2)	C8—C11—H11A	109.5
C1—N1—H1N	120.3 (18)	C8—C11—H11B	109.5
C1—N1—H2N	117.5 (18)	H11A—C11—H11B	109.5
H1N—N1—H2N	117.9 (14)	C8—C11—H11C	109.5
C7—N2—S1	126.54 (16)	H11A—C11—H11C	109.5
C7—N2—H3N	117.0 (18)	H11B—C11—H11C	109.5
S1—N2—H3N	116.4 (18)	C10—C12—H12A	109.5
C7—N3—C8	116.1 (2)	C10—C12—H12B	109.5
C7—N4—C10	116.50 (19)	H12A—C12—H12B	109.5
N1—C1—C2	121.3 (2)	C10—C12—H12C	109.5
N1—C1—C6	120.8 (2)	H12A—C12—H12C	109.5
C2—C1—C6	117.9 (2)	H12B—C12—H12C	109.5
C3—C2—C1	121.5 (2)	C18—C13—C14	119.9 (2)
C3—C2—H2	119.2	C18—C13—C19	119.4 (2)
C1—C2—H2	119.2	C14—C13—C19	120.6 (2)
C2—C3—C4	119.4 (2)	C15—C14—C13	119.7 (2)
C2—C3—H3	120.3	C15—C14—H14	120.2
C4—C3—H3	120.3	C13—C14—H14	120.2
C5—C4—C3	119.9 (2)	C14—C15—C16	120.2 (2)
C5—C4—S1	118.40 (17)	C14—C15—H15	119.9
C3—C4—S1	121.66 (17)	C16—C15—H15	119.9
C6—C5—C4	120.1 (2)	C15—C16—C17	120.3 (2)
C6—C5—H5	120.0	C15—C16—H16	119.8
C4—C5—H5	120.0	C17—C16—H16	119.8
C5—C6—C1	121.1 (2)	C18—C17—C16	119.5 (2)
C5—C6—H6	119.5	C18—C17—H17	120.2
C1—C6—H6	119.5	C16—C17—H17	120.2
N3—C7—N4	127.1 (2)	C17—C18—C13	120.3 (2)
N3—C7—N2	118.66 (19)	C17—C18—H18	119.8
N4—C7—N2	114.27 (19)	C13—C18—H18	119.8
N3—C8—C9	121.3 (2)	O3—C19—O4	123.3 (2)
N3—C8—C11	116.1 (2)	O3—C19—C13	122.3 (2)
C9—C8—C11	122.6 (2)	O4—C19—C13	114.41 (19)
			
O1—S1—N2—C7	47.5 (2)	S1—N2—C7—N3	5.5 (3)
O2—S1—N2—C7	174.35 (18)	S1—N2—C7—N4	−173.94 (16)
C4—S1—N2—C7	−70.9 (2)	C7—N3—C8—C9	0.6 (3)
N1—C1—C2—C3	179.8 (2)	C7—N3—C8—C11	−179.64 (19)
C6—C1—C2—C3	−2.1 (3)	N3—C8—C9—C10	−1.1 (3)
C1—C2—C3—C4	−0.2 (3)	C11—C8—C9—C10	179.1 (2)
C2—C3—C4—C5	1.7 (3)	C7—N4—C10—C9	1.1 (3)
C2—C3—C4—S1	−177.03 (17)	C7—N4—C10—C12	−179.1 (2)
O1—S1—C4—C5	145.66 (18)	C8—C9—C10—N4	0.2 (3)
O2—S1—C4—C5	13.6 (2)	C8—C9—C10—C12	−179.6 (2)
N2—S1—C4—C5	−97.07 (19)	C18—C13—C14—C15	0.5 (3)
O1—S1—C4—C3	−35.6 (2)	C19—C13—C14—C15	−177.2 (2)
O2—S1—C4—C3	−167.67 (17)	C13—C14—C15—C16	−0.6 (3)
N2—S1—C4—C3	81.6 (2)	C14—C15—C16—C17	0.6 (4)
C3—C4—C5—C6	−0.8 (3)	C15—C16—C17—C18	−0.4 (4)
S1—C4—C5—C6	177.91 (17)	C16—C17—C18—C13	0.2 (4)
C4—C5—C6—C1	−1.5 (4)	C14—C13—C18—C17	−0.2 (3)
N1—C1—C6—C5	−179.0 (2)	C19—C13—C18—C17	177.4 (2)
C2—C1—C6—C5	3.0 (3)	C18—C13—C19—O3	−3.6 (3)
C8—N3—C7—N4	0.9 (3)	C14—C13—C19—O3	174.0 (2)
C8—N3—C7—N2	−178.43 (19)	C18—C13—C19—O4	177.3 (2)
C10—N4—C7—N3	−1.7 (3)	C14—C13—C19—O4	−5.1 (3)
C10—N4—C7—N2	177.64 (19)		

### Hydrogen-bond geometry (Å, °)

**Table:**

Cg1 is the centroid of the C13–C18 ring.

**Table:**

*D*—H···*A*	*D*—H	H···*A*	*D*···*A*	*D*—H···*A*
O4—H4o···N4	0.847 (16)	1.793 (16)	2.639 (3)	177 (3)
N2—H3n···O3	0.885 (15)	1.904 (16)	2.787 (3)	176 (3)
N1—H1n···O1^i^	0.889 (18)	2.068 (18)	2.952 (3)	173 (3)
N1—H2n···O3^ii^	0.88 (2)	2.31 (3)	3.073 (3)	144 (2)
C12—H12c···O1^iii^	0.98	2.58	3.455 (3)	149
C11—H11c···Cg1^iv^	0.98	2.76	3.672 (3)	155

Symmetry codes:  (i) −*x*+3/2, *y*−1/2, *z*; (ii) −*x*+1, *y*−1/2, −*z*+3/2; (iii) *x*, −*y*+3/2, *z*−1/2; (iv) −*x*+1, −*y*+1, −*z*+1.
